# Wenlin procedure for chest wall reconstruction after tumor resection

**DOI:** 10.1093/jscr/rjac507

**Published:** 2022-11-11

**Authors:** Wenlin Wang, Weiguang Long, Yang Liu, Bin Cai, Juan Luo

**Affiliations:** Department of Chest Wall Surgery, Guangdong Second Provincial General Hospital, Guangzhou, China; Department of Chest Wall Surgery, Guangdong Second Provincial General Hospital, Guangzhou, China; Department of Chest Wall Surgery, Guangdong Second Provincial General Hospital, Guangzhou, China; Department of Chest Wall Surgery, Guangdong Second Provincial General Hospital, Guangzhou, China; Department of Chest Wall Surgery, Guangdong Second Provincial General Hospital, Guangzhou, China

## Abstract

After the resection of the chest wall tumor, there will be obvious defects in the chest wall, which needs to be reconstructed. In the past, reconstruction surgery mainly focused on the selection of materials rather than the surgical methods. Recently, We used Wenlin procedure to reconstruct the chest wall defect after tumor resection in a 65-year-old patient, and achieved satisfactory results.

## INTRODUCTION

Chest wall tumor is a common disease in chest wall surgery and generally needs to be removed [1, 2]. Because there will be residual defects in the chest wall after resection, reconstruction is required [3–5]. In the past, reconstruction surgery mainly focused on materials, and few people pay attention to the method of surgery [3–5]. Recently, we performed Wenlin procedure [6, 7] for reconstruction on a patient with chest wall tumor and achieved satisfactory results.

## CASE REPORT

**Figure 1:**
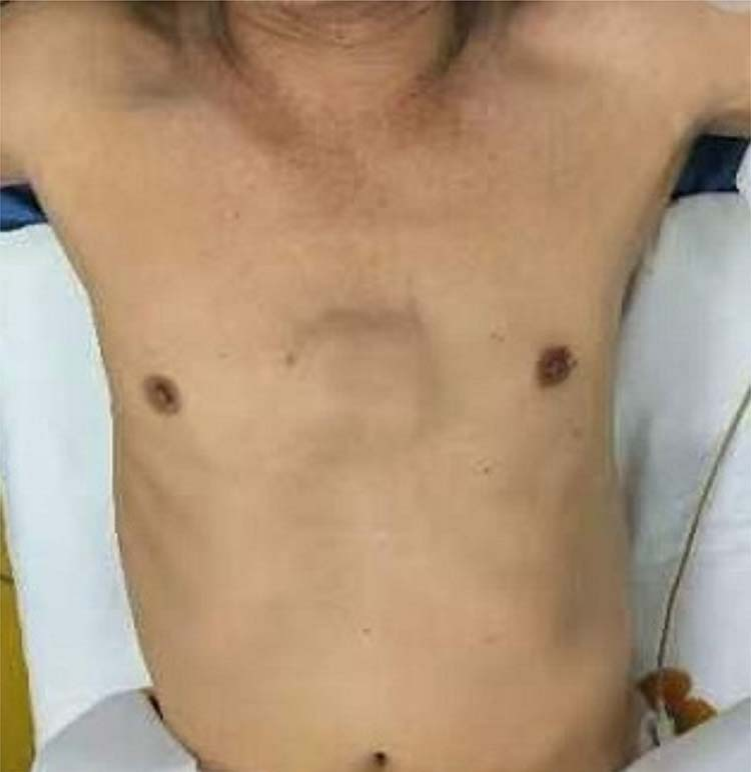
Location of tumor.

**Figure 2:**
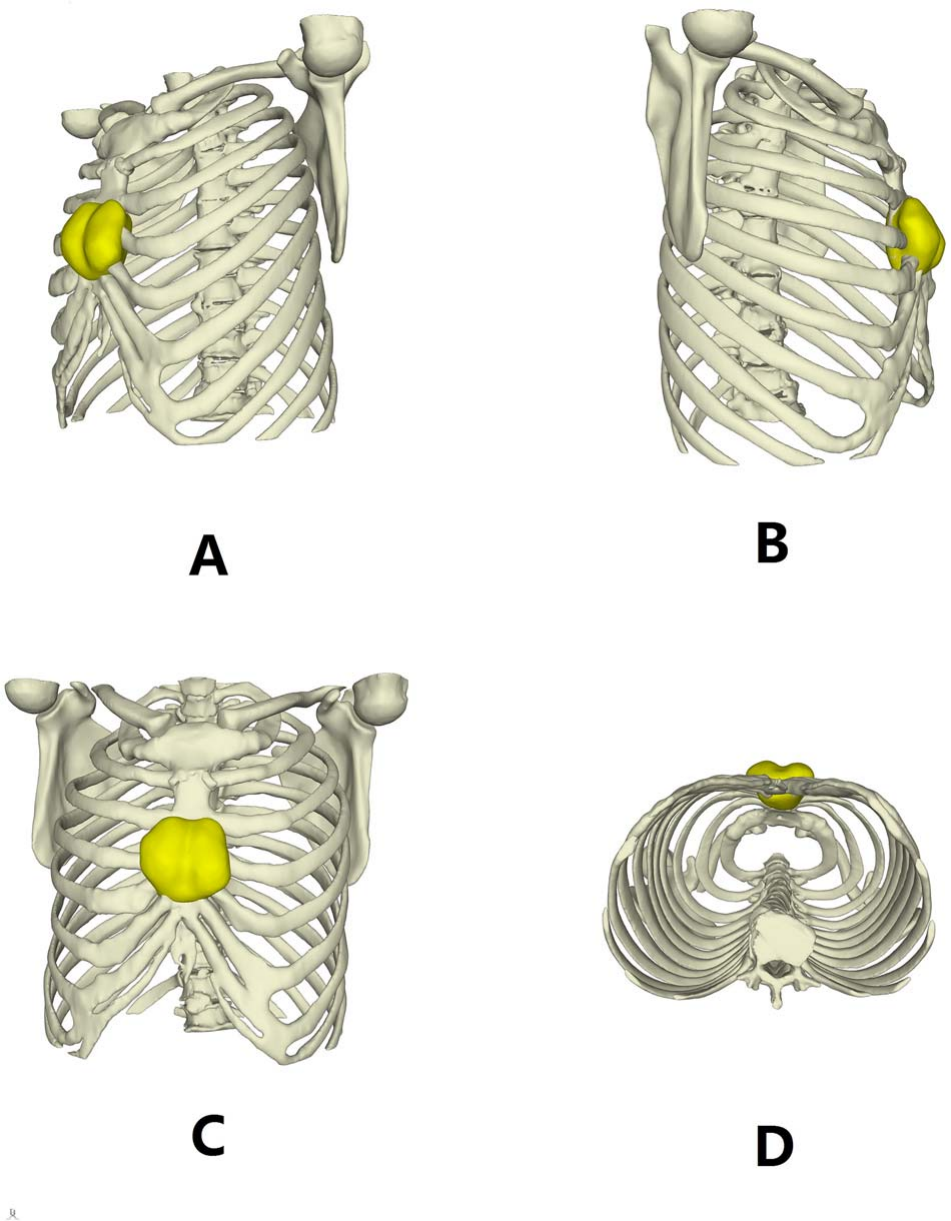
Three dimensional reconstruction images of the chest wall.

**Figure 3:**
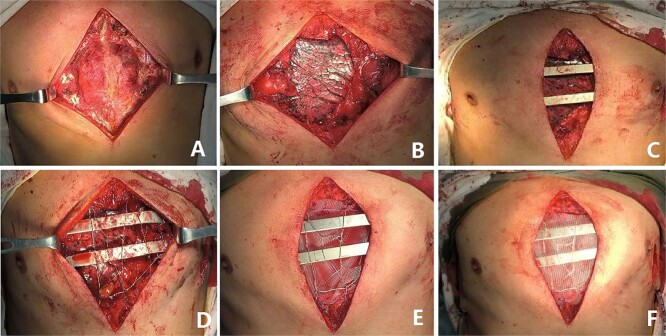
(**A**) Exposing tumor; (**B**) Resection of tumor; (**C**) Complete Wenlin procedure with two steel bars; (**D**) Wires mesh; (**E**), Fiber membrane is placed inside the steel bars and (**F**) The fiber membrane is placed outside the steel bars.

**Figure 4:**
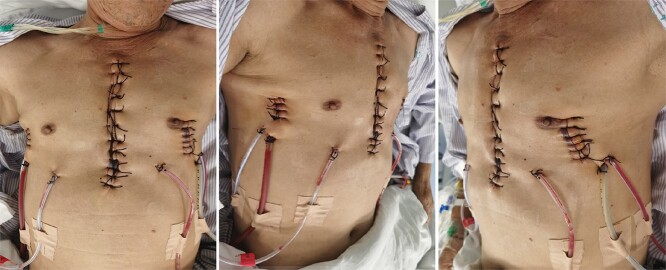
Appearance of chest wall after operation.

**Figure 5:**
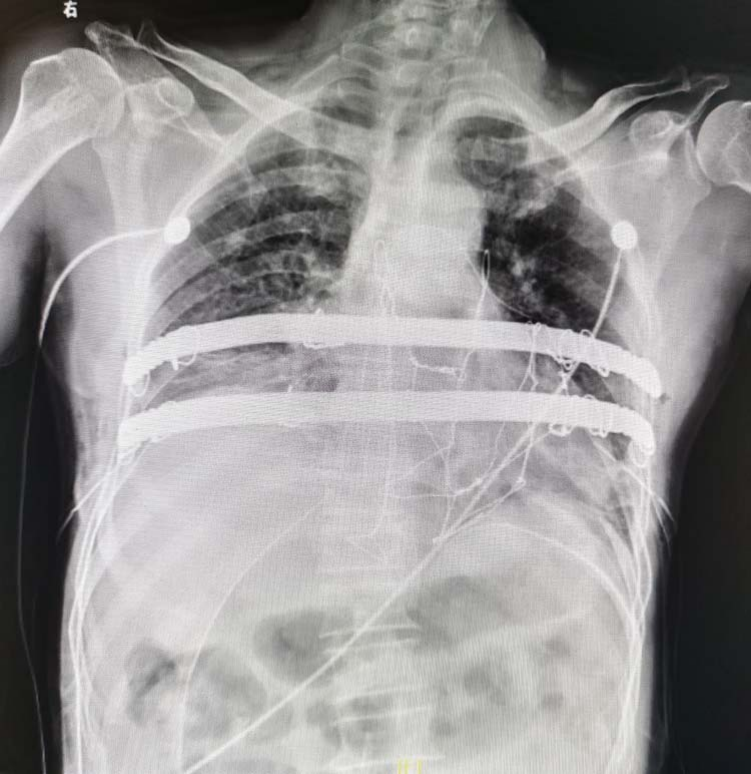
Postoperative X-ray examination.

The patient is a 65-year-old male. He was found to have a mass in the middle of the chest wall 1 year ago, without any discomfort. In the past 2 months, the mass grew rapidly with local pain. The patient was recently admitted to our hospital for surgery. Preoperative physical examination showed that there was a mass in the middle of the anterior chest wall, about 5 × 5 cm in size, with obvious tenderness and unclear boundary (Fig. 1). Imaging examination revealed a sternal tumor, which was located in the sternal body and invaded the surrounding costal cartilages (Fig. 2). The operation was performed under general anesthesia. In supine position, a longitudinal incision was made in the middle of the anterior chest wall to expose the tumor, and the tumor was removed along the periphery. The resection location was ~3 cm away from the border of tumor. After resection, a huge defect was formed in the middle of the anterior chest wall. Incisions were made on the lateral chest wall to expose the surrounding ribs. Wenlin procedure was performed with two steel bars [6, 7]. The curvature of the bar was the normal curvature of the chest wall. Both ends of the bar were firmly fixed with two adjacent ribs [8]. A mesh was woven between the two steel bars and the upper and lower ribs with steel wires, and the inner and outer sides of the steel bars were padded and fixed with fiber membranes. Drainage tubes were placed in both thoracic cavities and surgical fields (Fig. 3). After the incisions were closed, the operation was completed (Fig. 4). The operation time was 95 min, the intraoperative bleeding was 30 ml, and no complications occurred during the operation. The patient recovered smoothly after operation. Postoperative X-ray examination showed that the positions of the steel bars were normal (Fig. 5). He was discharged 10 days after operation.

## DISCUSSION

Chest wall tumor operation generally includes two parts, one is tumor resection, and the other is chest wall reconstruction [3–5]. Since the location of the tumor is superficial, and the resection is not difficult. The focus of the operation is considered to be chest wall reconstruction. In the past, the main point of reconstruction was the choice of materials, and no one paid much attention to the surgical method itself [3–5]. Considering the particularity of the chest wall itself, reconstruction should not only restore the integrity of the chest wall, but also obtain a normal appearance as far as possible. Therefore, the surgical method should also be emphasized.

Wenlin procedure is a surgical method for correcting thoracic deformity, which is especially suitable for correcting chest wall protrusion [6, 9–12]. Because the nature of this procedure is template plastic surgery, the shape of the chest wall can be restored to the maximum [1, 6, 7]. In Wenlin procedure, special steel bar is indispensable, which not only has certain elasticity, but also has great mechanical strength. Such physical properties make the bar also suitable for replacing general bone structures. It can be seen that Wenlin procedure can be used not only for the correction of deformities, but also for the reconstruction of the chest wall. We used this procedure in reconstructive surgery and achieved satisfactory results.

In order to make the chest wall more stable, we used a special fixation method to fix both ends of the steel bar during the reconstruction [8]. In addition, we also use steel wires to weave a mesh between the two steel bars and the surrounding ribs, which not only further strengthens the steel bars, but also eliminates the large gap between the bars. This will help eliminate possible paradoxical respiration after surgery. We used fibrous membranes to make cushions on the inside and outside of the steel bars, which is also conducive to eliminating a paradoxical respiration after surgery.

Our experience shows that Wenlin procedure is a reasonable choice for chest wall reconstruction. If there are suitable steel bars and correct techniques, the ideal effect may be obtained.
